# Primary Immature Teratoma of the Uterus Relapsing as Malignant Neuroepithelioma: Case Report and Review of the Literature

**DOI:** 10.1155/2013/971803

**Published:** 2013-06-04

**Authors:** Meryam Ben Ameur El Youbi, Amina Mohtaram, Jinane Kharmoum, Imane Aaribi, Saoussane Kharmoum, Abdelilah Bouzoubaa, Hind M'rabti, Saber Boutayeb, Basma El Khannoussi, Hassan Errihani

**Affiliations:** ^1^Department of Medical Oncology, National Institute of Oncology, Rabat, Morocco; ^2^Department of Pathology, National Institute of Oncology, Rabat, Morocco; ^3^Laboratory of Pathologic Anatomy and Cytology, Tangier, Morocco

## Abstract

*Background.* Although some mature cases of teratomas have recently been described in the cervix, they are not commonly found in the uterus, especially in immature forms. An immature uterine teratoma relapsing after surgery as malignant neuroepithelioma has never been reported in the literature. *Case Presentation.* We describe a case of immature teratoma which occurred primarily in the uterus in a 56-year-old female. Treatment consisted of total simple hysterectomy. Three months after surgery, the patient relapsed with voluminous pelvic mass and was treated with bleomycin, etoposide, and cisplatin-containing chemotherapy regimen. *Conclusion.* In this report and according to the pertinent literature, clinical and pathological features and management of uterine immature teratomas are discussed. The mainstay of treatment is surgery. The prognosis of this unusual disease remains uncertain.

## 1. Introduction

Teratomas are tumors commonly composed of multiple cell types derived from one or more of the three embryonic germ layers: ectoderm, endoderm, and mesoderm. These tissues are foreign to the location in which they are found. Teratomas may be classified as mature or immature on the basis of the presence of immature neuroectodermal elements within the tumor. Mature tumors have no immature elements. 

Teratomas are the most common germ cell tumors. They usually arise in the gonads and often occur in infancy and childhood. Extragonadal teratomas are rare and mainly develop in midline structures [1]. 

Primary teratomas of the uterus have rarely been reported since Mann's first description of this entity in 1929. Here we report a case of uterine immature teratoma in a 56-year-old woman which is an exceptional presentation of this tumor and review the relevant literature.

## 2. Case Presentation 

A 56-year-old Moroccan woman with no significant past medical history presented to gynecological consultation in June 2009 with a one-month history of dysuria, burning urination, and progressive lower abdominal distention. She had previously given birth to one child. The patient was menopausal since ten years ago.

Clinical examination revealed a painful distended abdomen with bulky uterus. The adnexa were normal on palpation.

Transabdominal ultrasonography demonstrated a solid uterine mass. The ovaries were not visualized and there was no ascite. Magnetic resonance imaging (MRI) of the pelvis showed a voluminous and heterogeneous uterine mass measuring 12 × 10 cm. The lesion was located at the uterine corpus and was displacing bladder and rectum. The ovaries appeared normal and there was no lymphadenopathy. The tumor appearance suggests a calcified fibroma.

The patient underwent a total simple hysterectomy. During surgery, a 12 cm mass was attached to the uterine corpus. The en bloc resection of this voluminous mass was impossible and the tumor was fragmented. Up to 20 pieces were removed. On sectioning, the fragments were macerated and showed various tissue consistencies.

**Figure 1:**
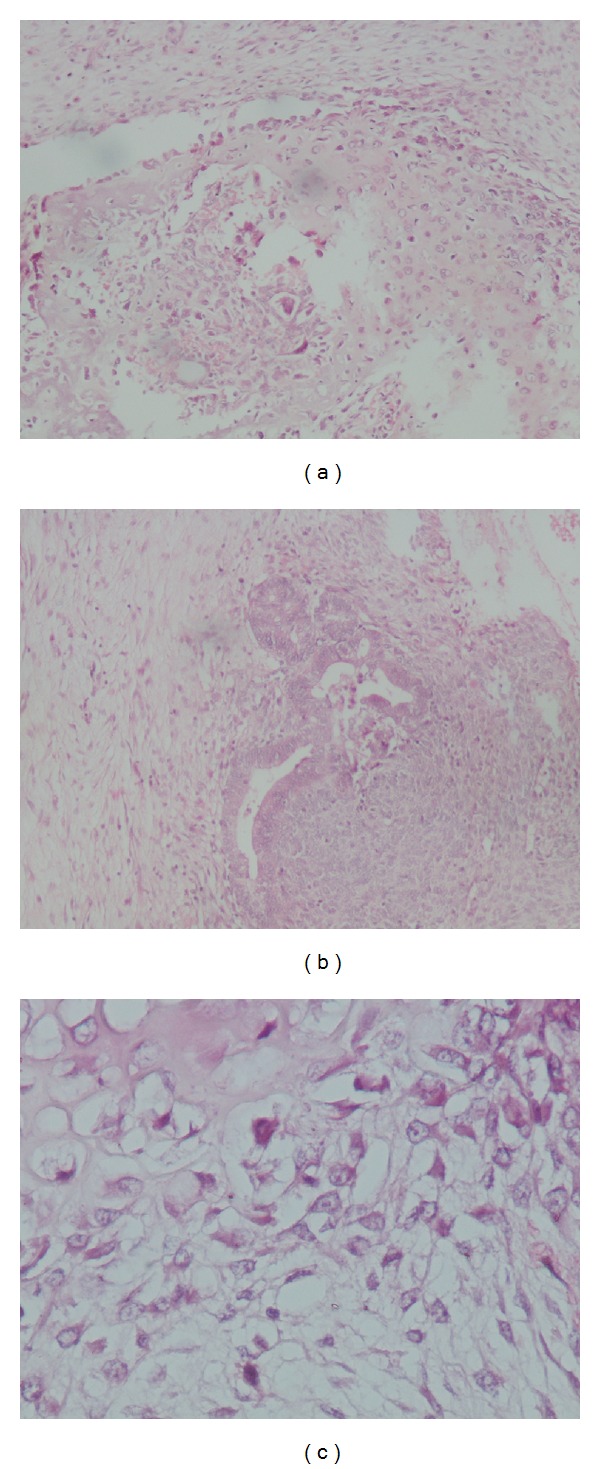
Microscopic findings of uterine mass specimen showing (a) cartilaginous foci (HES ×10), with (b) presence of glandular formations (HES ×20), and (c) ovoid cells involved in a fibrillar background (HES ×40).

Microscopically, the tumor was an immature teratoma, composed of a mixture of embryonic mature and immature elements. The tissue types found included nerve ovoid cells involved in a fibrillar background, active cartilaginous foci, and glandular formations. The immature component was dedifferentiated cells around epithelial tubes with the presence of mitoses (Figure 1). 

Our patient recovered well from surgery and a surveillance approach was taken. 

Three months later, the patient presented with vaginal bleeding. On digital examination of the vagina, she was found to have friable mass filling the upper half of the vagina with severe bleeding.

Pelvic and abdominal computer tomography (CT) scan revealed a bulky mass of 14 × 8 cm filling the pelvic cavity. The tumor was involving the bladder and the rectosigmoid with left ureterohydronephrosis. The scan of the thorax showed no evidence of extended disease.

Tumor markers including beta human chorionic gonadotropin (*β*hCG) and lactate dehydrogenase (LDH) were within normal limits. Alpha-fetoprotein (AFP) was raised at 556 ng/mL.

**Figure 2:**
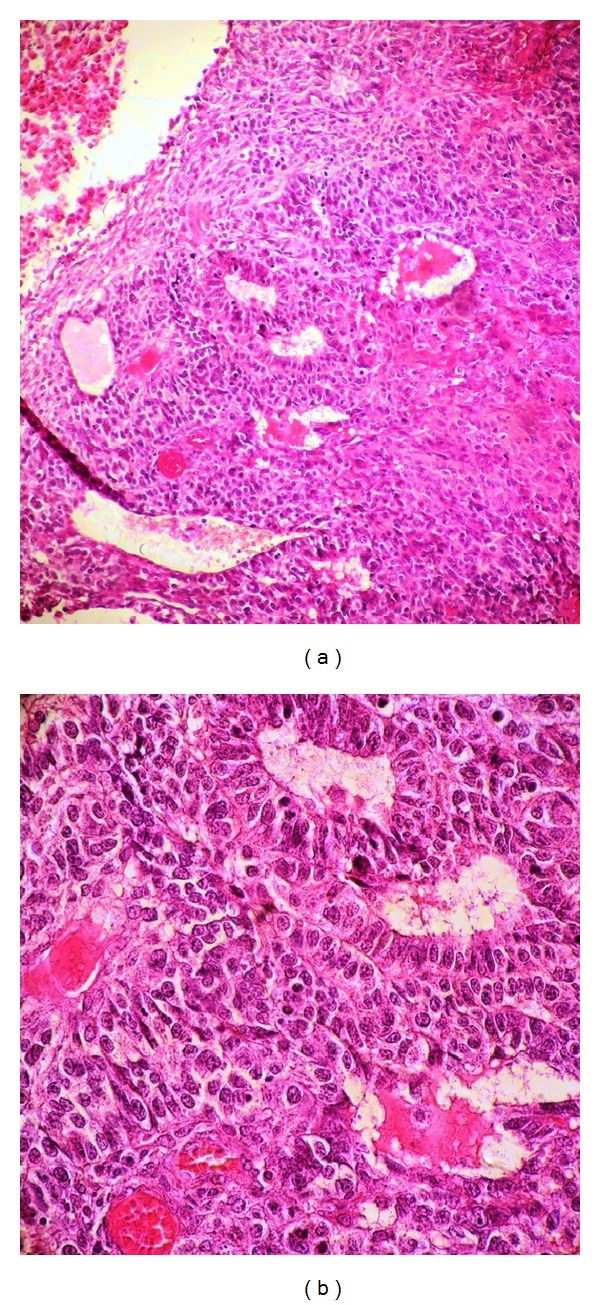
Histology of biopsy specimen revealing (a) diffuse sheets of neoplastic cells arranged in pseudorosettes and glandular formations (HES ×20), with (b) cytonuclear atypia and high mitotic index (HES ×40).

**Figure 3:**
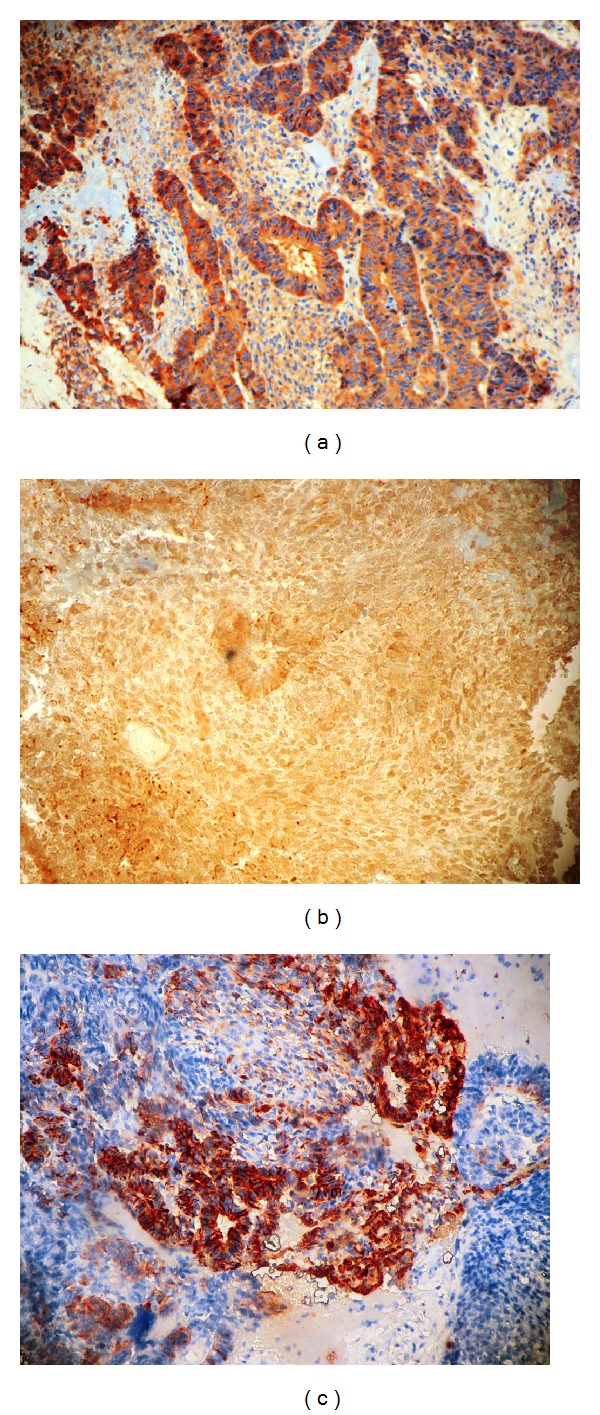
Immunohistochemical analysis showing positive staining for (a) NSE and (b) GFAP, with focal positive staining for (c) S100 protein.

Histological examination of the vaginal biopsy revealed a malignant embryonic proliferation with neuroepithelial component and mesenchymal areas, exhibiting high mitotic activity (Figure 2). Immunohistochemistry showed positive staining of the tumor cells for NSE and GFAP. Also, there was positive focal staining of tumor cells for S100 protein (Figure 3). A diagnosis of recurrence as a malignant neuroepithelioma of the immature teratoma was therefore made.

The treatment strategy included four courses of chemotherapy (BEP regimen) based on bleomycin (30 mg/day, days 1, 8, and 15), etoposide (100 mg/m²/day, days 1–5), and cisplatin (20 mg/m²/day, days 1–5) every 3 weeks, followed by surgical removing of residual mass. 

Unfortunately, our patient died when she returned home few days after the first course of chemotherapy.

## 3. Discussion

Extragonadal germ cell tumors are rare, comprising 1%-2% of all germ cell tumors. However, they can arise anywhere along the migration route of germ cells [2]. Extragonadal germ cells tumors usually develop in midline structures of the body including the mediastinum, retroperitoneum, thyroid, pericardium, pineal gland, and presacral region. Very rarely, other locations of those tumors such as parotid gland, breast, esophagus, stomach, liver, kidney, bladder, and uterine cervix have also been described but stay less common [3]. Also, their histological characteristics are similar to those of gonadal origin. 

Teratomas are the most common germ cell tumors. The occurrence of primary uterine teratoma is exceptional. In 1929, Mann was the first to describe a case of primary mature teratoma in the uterus [4]. Since then, only few cases have been reported. Histologically, the majority of the tumors were mature teratomas. To the best of our knowledge, only four cases of immature uterine teratoma have been described so far in the English literature (Table 1) [5–8]. 

Some interesting theories about the histogenesis of these tumors can be discussed. The first one suggests that uterine teratomas arise from primordial fetal germ cells that had abnormal migration from the fetal yolk sac endoderm to the gonadal ridge during early embryogenesis. As uterus is a midline organ, this supposes that primordial cells had a midline passage [5]. Another hypothesis proposes that residual fetal tissue can be implanted at any site of the genital canal during the fertility process [2, 5]. In our case, the patient was postmenopausal for several years; therefore, the possibility of implanted fetal tissue was ruled out. 

Uterine teratomas occur typically in the second to fourth decades of life. They may rarely occur in the case of postmenopausal women such as our patient. Clinically, there are no specific symptoms, and patients may present with abnormal vaginal bleeding, pelvic pain, or lower abdominal distention [5]. Pressure on the bladder with frequent or even obstructed urination can be noticed as well. 

A diagnosis of teratoma in the uterus cannot be made when imaging techniques do not display the characteristic findings. In CT or MRI, the appearance is variable with heterogonous, predominantly solid lesions or cystic or mixed solid and cystic lesions with small foci of fat or calcifications. The differential diagnosis might include uterine fibroid and lipoleiomyoma [9]. In previous reports and in the present case, radiological findings failed to make a correct preoperative diagnosis. 

Due to the extreme rarity of the immature uterine teratoma, there is no uniform consensus regarding treatment. However, the mainstay of treatment is surgery consisting of a complete tumor excision or radical hysterectomy with or without pelvic and para-aortic lymphadenectomy [2]. Also, the use of preoperative or postoperative chemotherapy can be extrapolated from ovarian malignant germ cells tumors guidelines [10]. In this case, three or four courses of BEP regimen are recommended [11]. As the benefit of adjuvant chemotherapy is not clearly established, surveillance can be a reasonable option [12]. Iwanaga et al. reported a case of 36-year-old woman, treated by surgery followed by two courses of VAC chemotherapy regimen without evidence of recurrence five years postoperatively [7]. In the paper published by Ansah-Boateng et al., the patient was treated by hysterectomy followed by pelvic radiotherapy; she has remained well with no evidence of recurrence [8]. Newsom-Davis et al. described chemotherapy for recurrent disease six months after surgery with etoposide cisplatin followed by paclitaxel etoposide and paclitaxel cisplatin, based on their institutional experience, and those alternating regimens have yielded no satisfactory response [5]. In the present case, systemic chemotherapy with BEP regimen was indicated in relapsed disease three months after prior surgery. However, chemotherapy response could not be assessed because of the patient death few days after starting the first course of BEP.

## 4. Conclusion

Immature uterine teratoma is an uncommon presentation for a uterine mass. Because of the extreme rarity of this disease, there is no therapeutic standard at the moment and the prognosis remains unknown.

## Figures and Tables

**Table 1:** Clinical characteristics and treatment of patients with primary immature teratoma of the uterus in the literature.

Author	Age	Symptoms	Site of tumor	Histology	Treatment	Relapse (months)	Treatment of relapse
Our case	56	Urinary symptoms Lower abdominal distention	Corpus	IT	Hysterectomy	Yes (3)	BEP × 1
Newsom-Davis et al., 2009 [5]	82	Vaginal bleeding	Corpus	IT	Hysterectomy and bilateral salpingooophorectomy	Yes (6)	EP × 1, ET × 1, TP × 1 + surgery
Gomez-Lobo et al., 2007 [6]	15	Vaginal bleeding Pelvic pain	ns	IT, high grade	Lesion excision + chemotherapy	ns	—
Iwanaga et al., 1993 [7]	36	Pelvic pain Lower abdominal distention	Fundus	IT, grade 3	Hysterectomy + chemotherapy (VAC × 2)	No	—
Ansah-Boateng et al., 1985 [8]	37	Vaginal bleeding	ns	IT, grade 2	Hysterectomy + radiotherapy	No	—

ns: not specified; IT: immature teratoma; VAC: vincristine, actinomycin D, cyclophosphamide; BEP: bleomycin, etoposide, cisplatin; EP: etoposide, cisplatin; ET: etoposide, paclitaxel; TP: palcitaxel, cisplatin.

## Abbreviations

MRI: Magnetic resonance imaging
CT: Computing tomography
*β*hCG: Beta human chorionic gonadotropin
LDH: Lactate dehydrogenase
AFP: Alpha-fetoprotein
NSE: Neuron-specific enolase
GFAP: Glial fibrillary acidic protein
BEP: Bleomycin, etoposide, cisplatin.
